# The complete mitochondrial genome of the Asian pitviper *Gloydius changdaoensis* (Squamata, Viperidae)

**DOI:** 10.1080/23802359.2020.1810154

**Published:** 2020-09-01

**Authors:** Yayong Wu, Ke Li, Qin Liu, Shanshan Chen, Bo Cai

**Affiliations:** a College of Life Science, Yibin University, Yibin, China; b Food Engineering, Shenyang Normal University, Shenyang, China; c Affiliated Traditional Chinese Medicine Hospital of Southwest Medical University, Luzhou, China; d Museum of Herpetology, Chengdu Institute of Biology, Chengdu, China

**Keywords:** Mitochondrial genome, phylogenetic, pitviper, snake, topotype

## Abstract

Asian pitviper *Gloydius changdaoensis* is a coastal species, distributed in Shandong province, China. In this study, we successfully sequenced the mitochondrial genome of one individual of *G. changdaoensis*. The complete mitochondrial genome is circular molecular with 17,224 bp, containing an origin of light-strand replication (OL), two non-coding control regions (CRs), and 37 classical genes of vertebrate, which contain 13 protein-coding genes (PCGs), 2 ribosomal RNA genes, and 22 transfer RNA genes. A Bayesian phylogenetic tree using the complete mitochondrial genomes of all viper species available showed a consistent result with previous studies.

*Gloydius* Hoge & Romano-Hoge, 1981 is a genus of venomous pitviper endemic to Asia. The specimens from Shandong peninsula, China, are considered by Li (1999) and Zhao (2006) that should be treated as a synonym or a subspecies of *G. saxatilis*, named *G. s. changdaoensis*. Based on the comparison of both mitochondrial gene fragments and morphology, Shi et al. (2016) stated that *G.s.changdaoensis* separated from *G. saxatilis*, and raised a valid species. However, the taxonomic status of this species is still controversial and undefined (Uetz et al. 2020).

In October 2018, a specimen (QY1213) of *G. changdaoensis* was collected from the type locality of Daheishan Island (37°58′N, 120°35′E), Changdao county, Shandong province, China, and used for sequencing of a complete mitogenome. Its liver tissue was fixed with 95% ethanol and stored at −20 °C in the herpetological collection, Chengdu Institute of Biology, Chinese Academy of Sciences. A small amount of liver tissue was shipped to Tsingke (Chengdu, China) for genomic extraction and 150-base-pair paired-end library construction; sequencing was performed on an Illumina HiSeq 2000 platform. De novo assembly of clean reads was performed using SPAdes v3.11.0 (Bankevich et al. 2012). Genes were annotated with the MITOS web server (Bernt et al. 2013), and then submitted to GenBank (accession no. MT731652). All sampling activities were conducted in accordance with the Guidelines of Animal Ethics published by the Chengdu Institute of Biology.

The complete length of the mitochondrial genomes (mitogenomes) of *G. changdaoensis* was 17,224 bp, containing an origin of light-strand replication (OL), two non-coding control regions (CRs), and 37 classical genes of vertebrate, which were 13 protein-coding genes (PCGs), two ribosomal RNA genes, and 22 transfer RNA genes. The base-pair composition was biased toward A and T with a 58.3% of A + T content on average (A 32.4%, C 28.7%, G 13.0%, and T 25.9%). The primary of the genes in the mitogenomes of *G. changdaoensis* were located on the heavy strand (H-strand) with except of one PCG (*ND6*) and eight tRNA genes (*tRNA-Gln, Ala, Asn, Cys, Tyr, Ser^UCN^, Glu,* and *Pro*). In 13 PCGs, the shortest was ATP8 gene (150 bp) and the longest was ND5 gene (1788 bp). Most PCGs initiated with ATG except for *COX1* with GTG, and *ND1* and *ND3* with ATA. Inverse, only five PCGs ended with complete stop codons, TAA (*ATP8, ATP6, ND4L, ND5,* and *Cyt b*), AGA (*COX1*), and AGG (*ND6*), and the other six genes ended with an incomplete stop codon, TTA (*ND1, ND2, COX2, COX3, ND3,* and *ND4*). The length of tRNA genes varied from 52 to 75 bp.

The concatenated PCGs were used to construct the Bayesian phylogenetic tree of *G. changdaoensis* and other 16 vipers. Bayesian phylogenetic tree indicated that all *Gloydius* were closely related to Crotalinae which were distributed in the America (PP 1.00) (Figure 1). Subsequently, all pit vipers of *Gloydius* appeared no less than two major phyletic lineages (PP 1.00). The taxa of *G. changdaoensis* was a sister relationship with a clade which was formed by *G. shedaoensis, G. saxatilis,* and *G. intermedius* (PP 1.00). It is evident that *G. changdaoensis* was a monophyly that was separated from *G. saxatilis* and *G. intermedius*.

**Figure 1.**
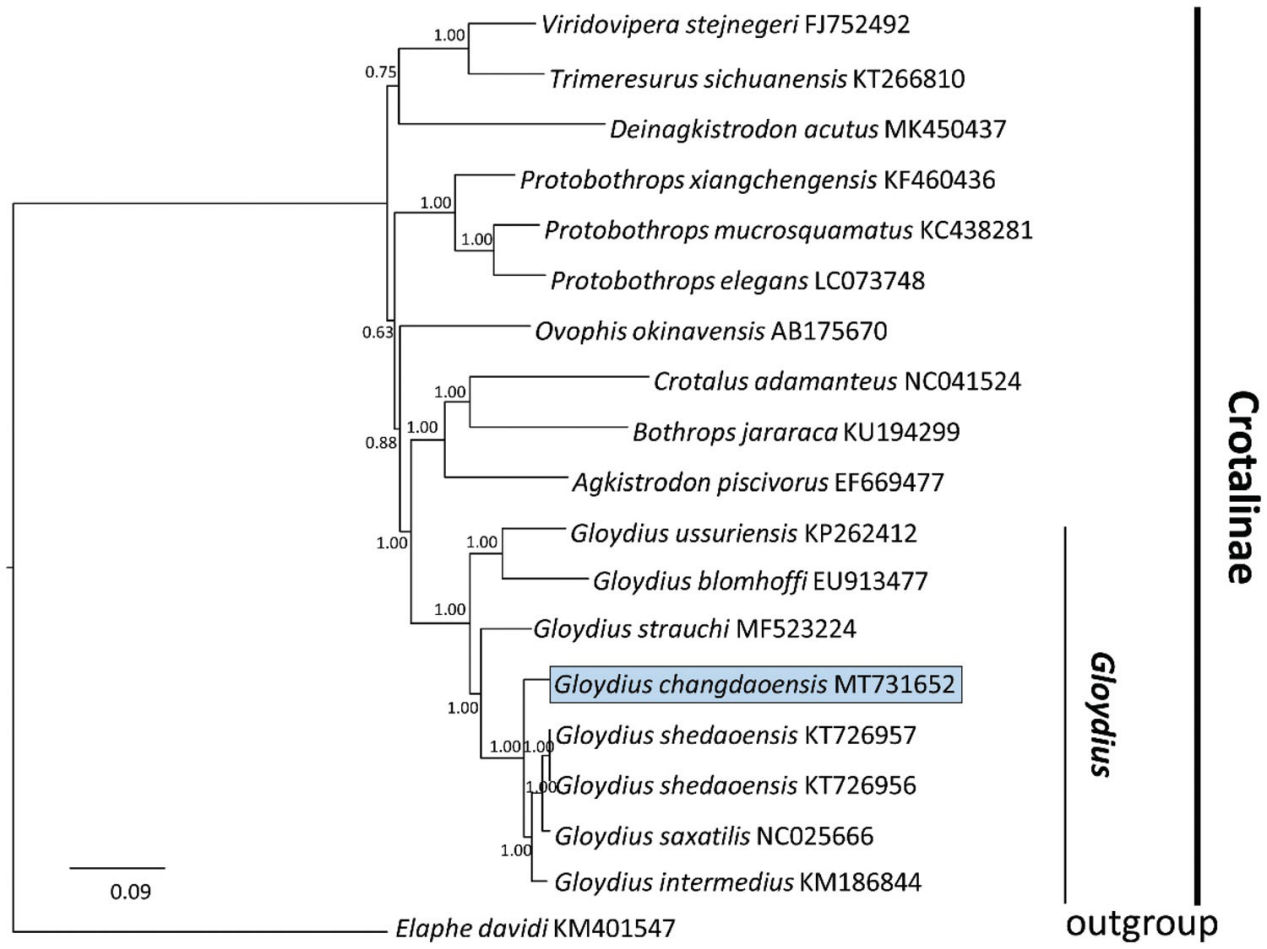
A majority-rule consensus tree inferred from Bayesian inference using MrBayes v.3.2.2 (Ronquist et al. 2012) with GTR + I+G substitution model that was selected by MrModelTest 2.3 under Akaike information criteria (Ronquist et al. 2012), based on the concatenated PCGs of 19 vipers of Crotalinae and one Colubridae taxa which were chosen as outgroup (Xu et al. 2016). DNA sequences were aligned in MEGA 7 (Kumar et al. 2016). Node numbers show Bayesian posterior probabilities. Branch lengths represent means of the posterior distribution. GenBank accession numbers were given with species names.

## Data Availability

The data that support the findings of this study are openly available in “NCBI” at https://www.ncbi.nlm.nih.gov/, reference number MT731652.
